# A Neutralizing Antibody Targeting Oxidized Phospholipids Promotes Bone Anabolism in Chow-Fed Young Adult Mice

**DOI:** 10.1002/jbmr.4173

**Published:** 2020-09-29

**Authors:** Michela Palmieri, Ha-Neui Kim, Horacio Gomez-Acevedo, Xuchu Que, Sotirios Tsimikas, Robert L Jilka, Stavros C Manolagas, Joseph L Witztum, Elena Ambrogini

**Affiliations:** 1 Division of Endocrinology and Metabolism, Center for Osteoporosis and Metabolic Bone Diseases and Center for Musculoskeletal Disease Research, University of Arkansas for Medical Sciences and Central Arkansas Veterans Healthcare System, Little Rock, AR, USA; 2 Department of Biomedical Informatics, University of Arkansas for Medical Sciences, Little Rock, AR, USA; 3 Division of Endocrinology and Metabolism, Department of Medicine, University of California San Diego, La Jolla, CA, USA; 4 Division of Cardiology, Department of Medicine, University of California San Diego, La Jolla, CA, USA

**Keywords:** OSTEOBLASTS, CELLS OF BONE, ANABOLICS, THERAPEUTICS, MOLECULAR PATHWAYS—REMODELING, BONE MODELING AND REMODELING

## Abstract

Oxidized phospholipids containing phosphocholine (OxPL) are pro-inflammatory lipid peroxidation products that bind to scavenger receptors (SRs), such as Scarb1, and toll-like receptors (TLRs). Excessive OxPL, as found in oxidized low-density lipoprotein (OxLDL), overwhelm these defense mechanisms and become pathogenic in atherosclerosis, nonalcoholic steatohepatitis (NASH), and osteoporosis. We previously reported that the innate IgM natural antibody E06 binds to OxPL and neutralizes their deleterious effects; expression of the single-chain (scFv) form of the antigen-binding domain of E06 (E06-scFv) as a transgene increases trabecular bone in male mice. We show herein that E06-scFv increases trabecular and cortical bone in female and male mice by increasing bone formation and decreasing osteoblast apoptosis in vivo. Homozygous E06-scFv mice have higher bone mass than hemizygous, showing a dose effect of the transgene. To investigate how OxPL restrain bone formation under physiologic conditions, we measured the levels of SRs and TLRs that bind OxPL. We found that osteoblastic cells primarily express Scarb1. Moreover, OxLDL-induced apoptosis and reduced differentiation were prevented in bone marrow-derived or calvaria-derived osteoblasts from Scarb1 knockout mice. Because Scarb1-deficient mice are reported to have high bone mass, our results suggest that E06 may promote bone anabolism in healthy young mice, at least in part, by neutralizing OxPL, which in turn promote Scarb1-mediated apoptosis of osteoblasts or osteoblast precursors.

## Introduction

Oxidation-specific epitopes (OSEs) are proinflammatory products of lipid peroxidation abundantly present on oxidized low-density lipoprotein (OxLDL) and the surface of apoptotic cells.^(1)^ OSE formation is greatly increased in the context of oxidative stress. OSEs are a class of danger associated molecular patterns (DAMPs) that are recognized and disposed of by innate immune pattern recognition receptors (PRRs), such as scavenger receptors (SRs), toll-like receptors (TLRs), and natural antibodies. A series of discoveries during the last 20 years has established that formation of OSEs is ubiquitous in inflammation and is a seminal pathogenic mechanism for atherosclerosis and nonalcoholic steatohepatitis (NASH), in which excessive OSEs accumulate relative to the capacity of the endogenous natural antibodies and scavenger receptors to dispose of them.^(2-4)^

Phospholipids containing the phosphocholine (PC) head-group are prominent components of all cell membranes, as well as low density lipoproteins (LDLs). Due to the presence of polyunsaturated fatty acids at the *sn-2* position, they are susceptible to reactive oxygen species (ROS)-mediated lipid peroxidation, forming PC-containing oxidized phospholipids (referred herein as OxPL). During the oxidation, the sn-2 fatty acid is modified by addition of one or more oxygen molecules and/or degraded to yield fragmented and shortened chains, some of which are reactive and lead to OxPL-protein adduct formation. As a result, although the PC is not itself affected by oxidative modifications, it is subjected to major conformational changes, rendering it more hydrophilic. Such OxPL are abundant in OxLDL and on the surface of apoptotic cells, but not native LDL or viable cells, and are ubiquitously present in inflammatory diseases (reviewed in Binder and colleagues^(2)^). The PC head group of OxPL, once exposed, is recognized by a number of innate pattern recognition receptors, including C-reactive protein (CRP), macrophage SRs, such as CD36 and Scarb1 (also known as SR-B1), and macrophage TLRs.^(2)^ Importantly, OxPL are also recognized by the innate IgM natural antibody E06, which binds to OxPL but not native phospholipids even though they have the PC headgroup. Similarly, E06 binds to OxLDL and apoptotic cells but not native LDL or viable cells. E06 blocks the pro-inflammatory properties of OxPL, in part by preventing their binding to SRs, TLRs, and other potential pro-inflammatory pathways.^(2)^ Importantly, to determine the role of OxPL in vivo, we previously generated transgenic mice expressing a single-chain variable fragment of E06 (E06-scFv). E06-scFv lacks an Fc effector domain of an antibody and, therefore, can only mediate its biological effects via binding to and neutralizing the effects of OxPL. Using the E06-scFv mice on the *Ldlr^−/−^* background, we showed that targeting OxPL profoundly decreased atherosclerosis in high cholesterol–fed mice, as well as NASH in murine models of diet-induced steatohepatitis, settings in which there is an abundance of OxPL.^(3,4)^ These beneficial effects resulted from inhibition of the uptake of OxLDL by macrophage SRs preventing foam cell formation, from decreasing macrophage-mediated inflammation and from improved hepatocyte mitochondrial function.^(3,4)^

We also previously showed that E06-scFv overexpression was protective against high fat diet-induced bone loss in *Ldlr^−/−^* mice.^(5)^ Unexpectedly, overexpression of E06-scFv in chow-fed C57BL/6J male mice also exhibited increased trabecular bone in vertebrae and in the distal femur.^(5)^ This bone anabolic effect was associated with increased bone formation, suggesting that chronic generation of OxPL, presumably as a consequence of “ambient” lipid peroxidation, restrains bone formation under physiologic conditions. The mice used for this earlier work were “homozygous” for the E06-scFv transgene, which is expressed under the control of the apolipoprotein E (ApoE) promoter; thus, both hepatocytes and macrophages contributed, leading to plasma levels of 20 to 30 μg/mL. Prompted by this preliminary evidence, we have investigated whether the bone anabolic effect of the E06-scFv transgene occurs in a dose-response manner in chow-fed mice, using homozygous and hemizygous mice of both sexes, and whether these changes are associated with increased bone formation. Because it is already known that osteoblasts express specific surface SRs and TLRs that can be activated by OxPL,^(2,6,7)^ we have also inquired if OxPL have direct effects on osteoblasts by these receptors.

We show here that the E06-scFv transgene has a profound dose-dependent bone anabolic effect on both trabecular and cortical bone in female as well as male mice fed a normal chow diet. These effects are associated with increased osteoblast number, increased rates of bone formation, and decreased osteoblast apoptosis in vivo. Moreover, OxPL promotes apoptosis and impairs the differentiation of osteoblasts in culture, and these effects are abrogated in osteoblasts lacking Scarb1.

## Subjects and Methods

### Animals

A detailed description of the E06-scFv transgenic mice has been published.^(3-5)^ Briefly, the E06-scFv transgene consists of a fusion protein of the heavy and light chain of the variable region domains of the E06 IgM, joined by a flexible peptide linker. The transgene is expressed under the control of the Apo-E promoter as described.^(3)^ Homozygous E06-scFv transgenic mice on C57BL/6J background were obtained from the University of California San Diego (UCSD) and maintained in our vivarium as hemizygous breeding pairs to obtain wild-type (WT), hemizigous, and homozygous littermates. The mice were genotyped by measuring the copy number of E06-scFv in tail extracts using qPCR of genomic DNA, as described.^(5)^ The copy number of hemizygous and homozygous mice was (mean ± SD) 60 ± 7 (range, 36–70) and 122 ± 15 (range, 82–140), respectively, in females, and 61 ± 8 (range, 43–79) and 115 ± 10 (range, 95–131), respectively, in males.

C57BL/6J (stock number 000664) and Scarb1 knock out mice on the C57BL/6J background (stock number 003379) were obtained from The Jackson Laboratory (Bar Harbor, ME, USA).

Mice were group-housed under specific pathogen-free conditions and maintained at a constant temperature of 23°C, in a 12:12-hour light-dark cycle; they had *ad libitum* access to chow (Teklad 22/5; 8640; Rodent Diet; Envigo, Somerset, NJ, USA) and water. To measure the bone formation rate, the mice were injected intraperitoneally with calcein 20 mg/kg body weight (Sigma-Aldrich, St. Louis, MO, USA) 7 and 3 days before harvest. Animals were euthanized by CO_2_ inhalation.

### Bone imaging

Determinations of skeletal architecture were performed using a micro-CT40 scanner (Scanco Medical AG, Bruettisellen, Switzerland). The fifth lumbar vertebra (L_5_), left femur, and right tibia were dissected and cleaned from muscles. The left femur was placed in 10% Millonig’s Neutral Buffered Formalin with 5% sucrose fixative (Leica Biosystems Inc., Buffalo Grove, IL, USA); L_5_ and right tibia were placed in B plus fixative (BBC Biomedical, Mount Vernon, WA, USA). After fixation, bones were dehydrated with ethanol and kept in 100% ethanol until analysis. For the micro-CT (μCT) scan, bones were loaded into 12.3-mm-diameter scanning tubes and imaged as described.^(5)^ Briefly, vertebrae, femurs, and tibias were scanned at 12 μm nominal isotropic voxel size, 500 projection (medium resolution, E = 55 kVp, I = 72 μA, 4 W, integration time 300 ms for femur and vertebra, 150 ms for tibia and threshold 200 mg/cm^3^), and integrated into three dimensional (3D) voxel images (1024 × 1024 pixel matrices for each individual planar stack). A Gaussian filter (sigma = 0.8, support = 1) was applied to all analyzed scans to reduce signal noise. During the conduct of these studies, the mean coefficient of variation of the μCT phantom was monitored weekly and was 1.23%. The entire vertebral body was scanned to obtain a number of slices varying between 270 and 300. Femurs were scanned from the distal epiphysis to the mid-diaphysis to obtain between 650 and 690 slices. Tibias were scanned from the proximal end of the tibia to the distal tibiofibular joint (TFJ), to obtain between 900 and 1000 slices.

For the μCT analysis, two-dimensional (2D) evaluation of trabecular bone was performed on contours of the cross sectional acquired images; primary spongiosa and cortex were excluded. In the vertebral body, contours were drawn from the rostral to the caudal growth plate to obtain 200 slices (12 μm/slice) and bone outside the vertebral body plate was excluded. The evaluation of the trabecular bone at the distal femur was performed on contours drawn on 151 slices (12 μm/slice), beginning eight to 10 slices away from the growth plate, to avoid the primary spongiosa, and proceeding proximally. The trabecular bone at the tibia was evaluated on contours drawn from the proximal end to the tibiofibular joint to obtain 100 slices (12 μm/slice). All trabecular measurements were performed on contours of the cross-sectional images drawn to exclude cortical bone and they were made every 10 to 20 slices. Voxel counting was used for bone volume per tissue volume measurements, and sphere-filling distance transformation indices were used for trabecular architecture with a threshold value of 220 mg/cm^3^, without preassumptions about the bone shape as a rod or plate.

Two dimensional evaluation of cortical bone was performed at mid-diaphysis in femur and tibia. In the femur, contours were drawn at mid-diaphysis (midpoint of the bone length as determined in scout view) to obtain 18 slices (12 μm/slice) with a threshold unit of 260 mg/cm^3^ for cortical thickness and −998 for endosteal and periosteal circumference. In the tibia, contours were drawn proximally to the distal tibiofibular joint (50–55 slices), to obtain 40 slices (12 μm/slice) with a threshold unit of 260 mg/cm^3^.

### Histomorphometry

L_1_–L_3_ vertebra were dissected and placed in 10% Millonig’s Neutral Buffered Formalin with 5% sucrose fixative, dehydrated with ethanol, and kept in ethanol 100% until analysis. Left femur was processed as described In the bone imaging section. Both vertebrae and femurs were embedded in methyl methacrylate (Sigma-Aldrich). Histomorphometric determinations were made as described.^(5)^ The analysis was done on 5-μm-thick longitudinal sections. Measurements were made on the trabecular bone of the vertebrae and the femurs. For the cortical measurements, both of the endosteal surfaces of a longitudinal section were analyzed. For measurement of static indices of osteoclast and osteoblast number, the sections were stained for tartrate-resistant acid phosphatase (TRAP) with toluidine blue counterstaining. Osteoblasts were identified as teams of cells (≥2) overlying osteoid. The osteoblasts and osteoclast numbers are reported over bone perimeter. Those measurements were performed in a blinded fashion using Osteomeasure version 7 V4.3.0.0 (OsteoMetrics, Inc., Decatur, GA, USA). Unstained sections were used for the determination of fluorescent calcein labeling using fluorescence microscopy. Bone formation rate (BFR) is defined as the distance between the double labels divided by the interval between the fluorochrome administrations and multiplied by the sum of the double-labeled perimeter and one-half of the single-labeled perimeter. If only a single label was present, data were treated as missing values for statistical purposes. Those measurements were performed using Osteomeasure version XP 3.1.0.1 (OsteoMetrics, Inc.). Histomorphometric data are reported using the nomenclature recommended by the American Society for Bone and Mineral Research.^(8)^ The imaging were obtained with Universal Microscope Axioplan 2 (Zeiss, Inc., Thornwood, NY, USA) using an Olympus DP74 camera and Olympus cellSens Entry 2.1 acquisition software (Olympus, Waltham, MA, USA).

### Measurement of lean and fat body mass

The percentages of lean and fat body mass were calculated by dual-energy X-ray absorptiometry (DXA) of sedated mice (2% isoflurane) using a PIXIimus densitometer (GE Lunar, Madison, WI, USA). The mean coefficient of variation of the percentage of body fat of a proprietary phantom (performed prior to each use) during the conduct of these studies was 0.5.

### Quantification of osteoblast apoptosis in vivo

In vivo osteoblast apoptosis was quantified by using the Dead-End™ Fluometric TUNEL System (Promega, Madison, WI, USA) according to the manufacturer’s instructions. Briefly, vertebral sections embedded in methyl methacrylate were deplastified using xylene and monoethanolamine. The slides were washed with acetone, rehydrated with decreasing concentrations of ethanol, and rinsed in distilled water. The slides were then fixed in 4% formaldehyde, permeabilized with proteinase K solution (20 μg/mL), fixed again and equilibrated with equilibration buffer (100 μL). The samples were then labeled with terminal deoxynucleotidyl transferase (TdT) and incubated for 60 min in a humidified chamber and then counterstained with DAPI. The apoptotic osteoblasts were identified by green flourescence using confocal fluorescence microscopy (Zeiss LSM 880 laser confocal microscope; Zeiss, Inc.) using the following settings: objective = EC plan-Neofluar 40×/1.3 oil; wavelength of excitation 405 nm for DAPI and 488 nm for TUNEL staining. We obtained 25 tiled images (sample in five-frame stack with 3.5-μm interval). The stacks were converted to orthogonal projections and the osteoblasts were counted using Zen 2.3 software (Zeiss, Inc.).

### Culture of osteoblastic cells

Calvaria cells were isolated from neonatal pups (either C57BL/6J or Scarb1 knockout mice) by sequential digestion with collagenase type 2 (Worthington, Columbus, OH, USA; CLS-2, lot 47E17554B) as described.^(9)^ Bone marrow cells were obtained by flushing the femoral diaphysis (after removing the proximal and distal ends) with α-MEM medium (Invitrogen, Carlsbad, CA, USA). Bone marrow stromal and calvaria cells were cultured in α-MEM containing 10% Premium Select fetal bovine serum (FBS) (Atlanta Biologicals, Flowery Branch, GA, USA), 1% penicillin/streptomycin/glutamine (PSG) in presence of 1mM ascorbate-2-phosphate up to 70% confluence. Bone marrow cells from E06-scFv homozygous and hemizygous transgenic mice and their WT littermates were cultured in α-MEM containing 10% Premium Select FBS and 1% PSG in presence of 1mM ascorbate-2-phosphate and 10mM beta-glycerophosphate for 10 days. OB6,^(10)^ MC3T3-E1,^(11)^ and UAMS-32^(12)^ cells were cultured in α-MEM containing 10% Premium Select FBS and 1% PSG until 80% confluence. MLO-Y4^(13)^ were cultured in α-MEM containing 5% Premium Select FBS, 5% bovine calf serum (HyClone Laboratories, Logan, UT, USA), and 1% PSG until 80% confluence.

### Osteoclast differentiation

Osteoclasts were cultured as described.^(14)^ Briefly, whole bone marrow cells were cultured with 10% FBS, 1% PSG for 24 hours in the presence of 10 ng/mL M-CSF (R&D Systems, Minneapolis, MN, USA). Nonadherent bone marrow cells were then cultured with 30 ng/mL of M-CSF and 30 ng/mL of RANKL (R&D Systems) for 5 days. After fixation with 10% neutral buffered formalin for 10 min, the cells were stained for TRAP, using the Leukocyte Acid Phosphatase Assay Kit, following the manufacturer’s instructions (Sigma-Aldrich). An osteoclast was defined as a multinuclear TRAP-positive cell.

### qPCR

Total RNA was extracted from cells and tissues with Trizol (Thermo Fisher Scientific, Waltham, MA, USA) and purified with Direct-zol RNA miniprep (Zymo Research, Irvine, CA, USA; cat no. R2050). cDNA was reverse transcribed from 0.5 μg of total RNA extract using the High-Capacity cDNA Reverse Transcription kit (Applied Biosystems, Foster City, CA, USA) according to the manufacturer’s instructions. TaqMan PCR was performed using TaqMan Gene Expression Assays manufactured by Applied Biosystems, as listed in Supplementary Table 6. Transcript levels were calculated by normalizing to the reference gene mitochondrial ribosomal protein S2 using the delta threshold cycle (ΔCt) method.^(15)^

### Gene expression silencing

To silence the expression of Scarb1, we used a short hairpin RNA lentiviral infection system (Sigma-Aldrich) according to the manufacturer’s instructions. In brief, we incubated newborn calvaria cells with the lentiviral particles (#SHCLNV) in the presence of hexadmetrine bromide (8 μg/mL) (Sigma-Aldrich). Non-targeted shRNA lentiviral particles (#SHC001V) were used as a scrambled shRNA sequence control. We further cultured the infected cells with puromycin at the concentration of 1 μg/mL (Santa Cruz Biotechnology, Dallas, TX, USA) for 72 hours to remove uninfected cells.

### Capsase 3 activity

Apoptotic cells were quantified by measuring caspase 3 activity in cell lysates as described.^(16)^ Caspase 3 activity was measured by determining the degradation of the fluorometric substrate DEVD-AFC, which contains the amino acid sequence of the caspase 3 cleavage site DEVD (Asp-Glu-Val-Asp), conjugated with 7-amino-4-trifluoromethylcoumarin (AFC). Briefly, lysates (100 μg protein) were incubated with 50μM DEVD-AFC in 50mM zwitterionic sulfonic acid (HEPES) (pH 7.4), 100mM NaCl, 0.1% 3- ((3-Cholamidopropyl) dimethylammonio)-1-Propanesulfonic Acid (CHAPS), 10mM dithiothreitol (DTT), 1mM EDTA, and 10% glycerol. The released fluorescent AFC was measured in a microplate fluorescence reader FL500 (BioTek Instruments, Winooski, VT, USA) with excitation/emission wavelengths of 400/510 nm. Protein concentration in the lysate was measured using a Bio-Rad detergent–compatible kit (Bio-Rad Laboratories, Hercules, CA, USA). OxLDL was obtained from Alfa Aesar (Alpha Aesar, Haverhill, MA, USA) and E06 IgM from AVANTI Polar Lipids Inc. (Alabaster, AL, USA; Cat Number 330001-100 μg, lot number E06-17 and E06-19).

### Alkaline phosphatase activity

For the alkaline phosphatase (ALP) activity measurement, the cells were lysed in 100mM glycine, 1mM MgCl_2_, and 1% Triton X-100 at pH 10 using a buffer containing 2-amino-2-methylpropanol and p-nitrophenylphosphate (Sigma-Aldrich). ALP activity was normalized to total protein concentration measured using Bio-Rad detergent-compatible kit (Bio-Rad Laboratories). For all assays, triplicate cultures were analyzed.

### ELISA

The binding titer of active E06-scFv, which contains a His tag, was measured in the serum as described using a phosphorylcholine conjugated to bovine serum albumin (PC-BSA)-coated microtiter plate.^(3,5)^ To measure the total level of E06-scFv monovalent and immune complexes in the serum, 96-well round-bottomed microplates were coated with the anti-T15/E06 idiotype antibody AB1-2 in PBS (5 μg/mL) overnight at 4°C. The plates were then washed with PBS three times, blocked with 1% BSA in PBS (75 μL per well) for 45 min at room temperature, and then washed again three times with PBS. Serum from homozygous or hemizygous E06-scFv transgenic mice was diluted 1:40 with 1% BSA-PBS and incubated for 90 min at room temperature. Bound E06-scFv was detected with anti-His tag antibody conjugated with ALP (SouthernBiotech, Birmingham, AL, USA; catalog number 4603-04), diluted in Tris buffered saline (TBS) buffer containing 1% BSA (1:1000 dilution, 50 μL per well) and incubated for 60 min at room temperature. The plates were then washed with PBS and rinsed with deionized water to remove phosphates, and then 25 μL of 50% (1:1 in distilled H_2_O) LumiPhos 530 (Lumigen, Southfield, MI, USA) as luminescent substrate was added. Chemiluminescence was measured as relative light units (RLU) over 100 ms using a plate Luminometer (BioTek Synergy HTX Multi-Mode Reader; BioTek Instruments). The RLU were normalized to a reference standard pool of sera from hemizygous mice that was used in every plate; to do that we divided the RLU from the sample by the RLU from the standard. All determinations were done in two separate assays and in triplicate.

### Statistics

**Fig 1.**
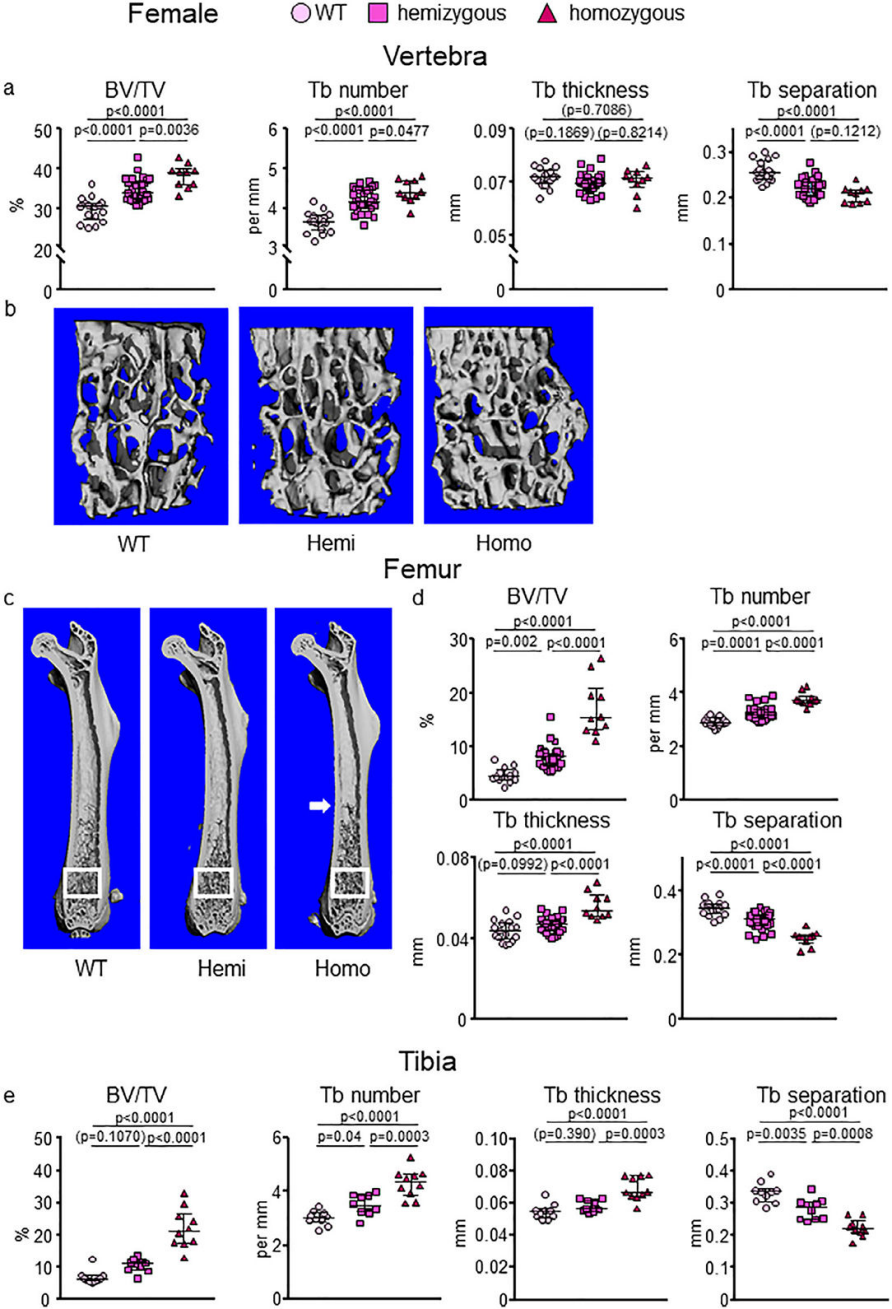
E06-scFv increases trabecular bone at the spine, femur, and tibia in E06-scFv transgenic female mice. μCT determination of (*A,B*) vertebral, (*C,D*) femoral, and (*E*) tibial trabecular bone architecture in 6-month-old WT, hemizygous, and homozygous E06-scFv transgenic female mice. (*A*) Quantification of vertebral trabecular bone architecture. WT *n* = 17; hemizygous E06-scFv *n* = 32; homozygous E06-scFv *n* = 10. (*B*) Lateral images of vertebral trabecular bone (one representative image was chosen for each group). (*C*) Longitudinal images of femur, with proximal end at the top (one representative image was chosen for each group). White boxes indicate the area where trabecular bone was quantified. (*D*) Quantification of femoral trabecular bone architecture in the same mice as in *A*. WT *n* = 17; hemizygous E06-scFv *n* = 31; homozygous E06-scFv *n* = 10. (*E*) Quantification of tibial trabecular bone architecture in a subgroup of the mice shown in *A* and *B*. WT *n* = 10; hemizygous E06-scFv *n* = 10; homozygous E06-scFv *n* = 10. Data are shown as individual values and median with interquartile range. Data analyzed by ANOVA, the *p* values shown are adjusted using the Tukey’s pairwise comparison procedure. BV/TV = bone volume /total volume; Tb = trabecular; WT = wild-type.

**Fig 2.**
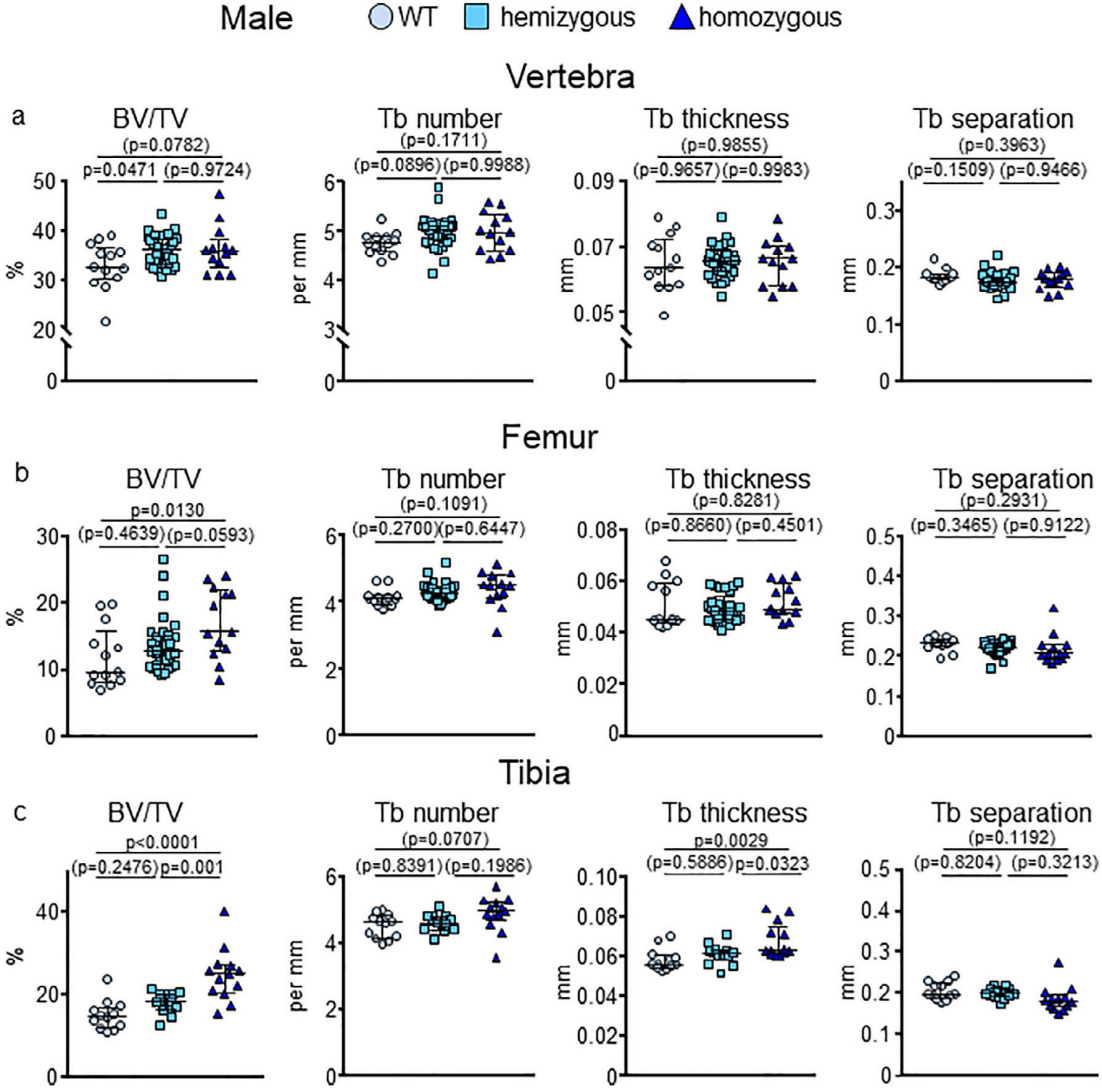
E06-scFv increases trabecular bone at the spine, femur, and tibia in E06-scFv transgenic male mice. μCT determination of (*A*) vertebral, (*B*) femoral, and (*C*) tibial trabecular bone architecture in 6-month-old WT, hemizygous, and homozygous E06-scFv transgenic male mice. (*A*) Quantification of vertebral trabecular bone architecture. WT *n* = 13; hemizygous E06-scFv *n* = 34; homozygous E06-scFv *n* = 13. (*B*) Quantification of femoral trabecular bone architecture in the same mice as in *A*. WT *n* = 13; hemizygous E06-scFv *n* = 33; homozygous E06-scFv *n* = 13. (*C*) Quantification of tibial trabecular bone architecture in a subgroup of the mice shown in *A* and *B*. WT *n* = 12; hemizygous E06-scFv *n* = 13; homozygous E06-scFv *n* = 13. Data are shown as individual values and median with interquartile range. Data analyzed by ANOVA, the *p* values shown are adjusted using the Tukey’s pairwise comparison procedure. BV/TV = bone volume/total volume; Tb = trabecular.

**Fig 3.**
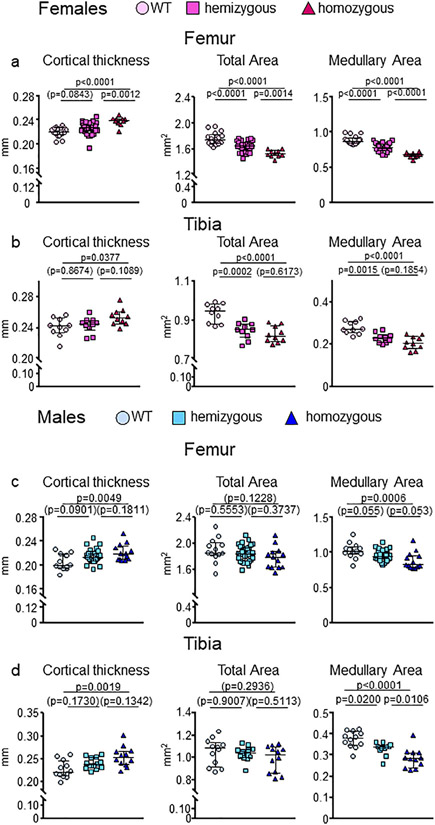
E06-scFv increases cortical thickness in both femur and tibia in female and male E06-scFv transgenic mice. (*A*) μCT determination of femoral cortical bone architecture in 6-month-old WT, hemizygous, and homozygous E06-scFv transgenic female mice. WT *n* = 17; E06-scFv hemizygous *n* = 31; E06-scFv homozygous *n* = 10. (*B*) μCT determination of tibial cortical thickness of a subgroup of the female mice shown in *A*. WT *n* = 10; hemizygous E06-scFv *n* = 10; homozygous E06-scFv *n* = 10. (*C*) μCT determination of femoral cortical bone architecture in 6-month-old male WT, hemizygous, and homozygous E06-scFv transgenic male mice. WT *n* = 13; hemizygous E06-scFv *n* = 33; homozygous E06-scFv *n* = 13. (*D*) μCT determination of tibial cortical thickness of a subgroup of the male mice shown in *C*. WT *n* = 12; hemizygous E06-scFv *n* = 13; homozygous E06-scFv *n* = 12. Data are shown as individual values and median with interquartile range. Data analyzed by ANOVA, the *p* values shown are adjusted using the Tukey’s pairwise comparison procedure.

**Fig 4.**
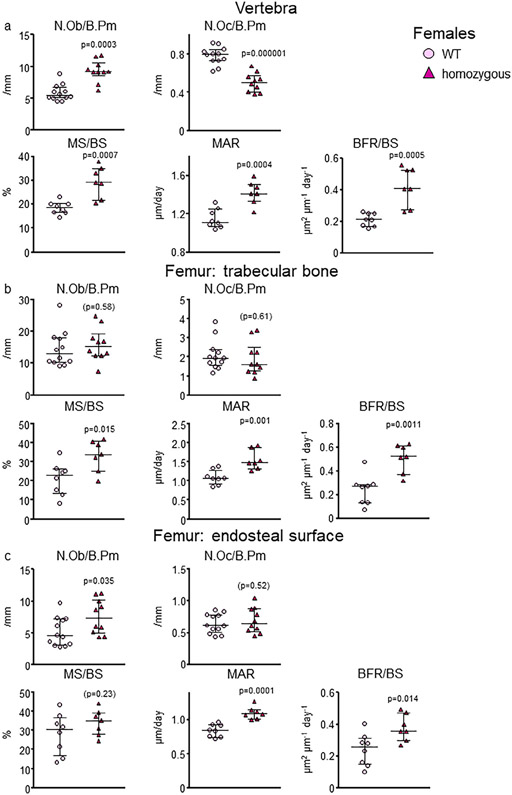
E06-scFv affects bone mass by increasing osteoblasts in trabecular and cortical bone. (*A*) Histomorphometry of the trabecular vertebral bone surface in WT and E06-scFv homozygous transgenic female mice. Osteoblast and osteoclast numbers: WT *n* = 12; homozygous E06-scFv *n* = 10. MS/BS, MAR, and BFR/BS: WT *n* = 8; homozygous E06-scFv *n* = 7. (*B*) Histomorphometry of the trabecular femoral bone surface in WT and E06sc-Fv homozygous transgenic female mice. Osteoblast and osteoclast numbers: WT *n* = 11; homozygous E06-scFv *n* = 10. MS/BS, MAR, and BFR/BS: WT *n* = 8 and homozygous E06-scFv *n* = 7. (*C*) Histomorphometry of the endosteal surface of the cortical femoral bone in WT and E06sc-Fv homozygous transgenic female mice. Osteoblast and osteoclast numbers: WT *n* = 12; homozygous E06-scFv *n* = 10. MS/BS, MAR, and BFR/BS: WT *n* = 8; homozygous E06-scFv *n* = 7. Data are shown as individual values and median with interquartile range. Data analyzed by Student’s *t* test. B.Pm = bone perimeter; BFR = bone formation rate; BS = bone surface; MAR = mineral apposition rate; MS = mineralized surface; Ob.N = osteoblast number; Oc.N = osteoclast number.

No experimentally derived data were excluded. In Figs. 1, 2, and 3, two femurs, one per each sex in the transgenic E06-scFv hemizygous group, and in Fig. 3D one tibia in the homozygous male group, could not be analyzed with μCT because they were damaged during the harvest. In Fig. 4, poor section quality precluded the histological analysis for the bone formation rate of some samples and the numbers of samples analyzed are specified in the figure legend. In Supplementary Fig. 1b,d, the femoral length could not be measured in one E06-scFv hemizygous and one WT female, and two hemizygous and one homozygous E06-scFv males, because the femur was damaged at either the proximal or the distal end.

**FIGURE 5:**
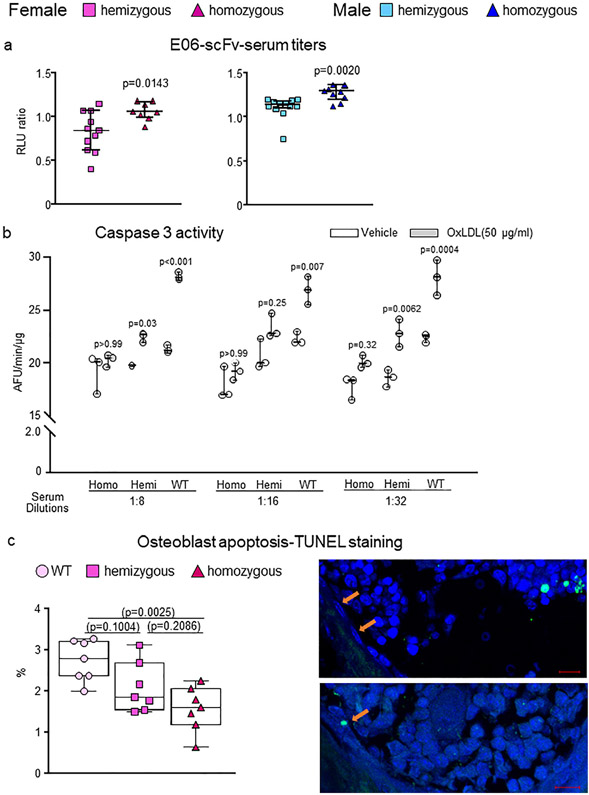
Serum from E06-scFv homozygous and hemizygous mice prevents the pro-apoptotic effect of OxLDL. (*A*) Relative levels of E06-scFv in the serum of 6-month-old hemizygous or homozygous female and male transgenic mice determined by ELISA. Female: hemizygous E06-scFv *n* = 11; homozygous E06-scFv *n* = 8. Male: hemizygous E06-scFv *n* = 12; homozygous E06-scFv *n* = 10. E06-scFv was undetectable in serum of WT mice (not shown). Data analyzed by *t* test. Data are shown as individual values and median and interquartile range. (*B*) Osteoblastic cells obtained from WT neonatal calvaria were pretreated for 1 hour with serum from hemizygous or homozygous E06-scFv transgenic or C57BL/6J (WT) female mice at the indicated dilutions (1:8, 1:16, 1:32). Serum from 10 mice per genotype was pooled for this analysis. Caspase 3 activity was measured 6 hours after addition of vehicle or OxLDL (50 μg/mL). All measures were performed in triplicate cultures. Data for each dilution were analyzed by one-way ANOVA with Bonferroni correction; the *p* values reflect comparison to the respective vehicle. Data are shown as box plots with individual values and median with interquartile range. (*C*) Quantification of osteoblasts apoptosis in vivo by TUNEL staining in vertebrae of WT, hemizygous, and homozygous female mice (*n* = 7/group). Results are expressed as percentage of apoptotic osteoblasts over total number of osteoblasts counted in the section. Data are shown as box-plots with individual values and median with interquartile range. Representative imaging of vertebral longitudinal section stained with DAPI and TUNEL. Non-apoptotic osteoblasts are indicated on the top figure and an apoptotic osteoblast is indicated on the bottom figure (scale = 10 μm). Data analyzed by ANOVA, the *p* values shown are adjusted using the Tukey’s pairwise comparison procedure. AFU = Arbitrary fluorescence units.

**Fig 6.**
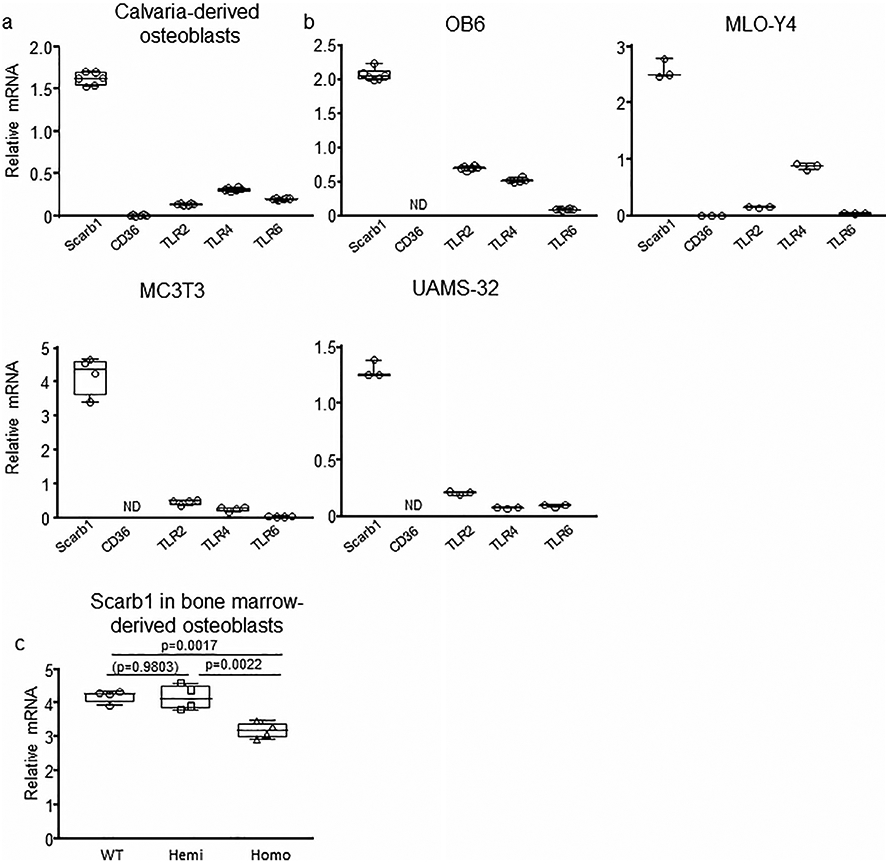
Scarb1 is the most abundant scavenger receptors in osteoblasts and His levels decline in homozygous E06-scFv mice. The expression of indicated scavenger receptors (CD36 and Scarb1) and TLRs known to bind to PC-OxPL were quantified with RT-PCR in (*A*) calvaria-derived osteoblasts and (*B*) the indicated osteoblast/osteocyte cell lines. Data are shown as box-plots with individual values and median with interquartile range. (*C*) Expression of Scarb1 in bone marrow-–derived osteoblasts from 4-month-old to 5-month-old WT, hemizygous, and homozygours E06sc-Fv transgenic mice, cultured for 10 days in presence of 1mM ascorbate-2-phosphate and 10mM beta-glycerophosphate. Target gene expression was calculated by normalizing to ChoB. Data are shown as box plots with individual values and median with interquartile range. Data analyzed by ANOVA, the shown p values are adjusted using the Tukey’s pairwise comparison procedure. All measures were performed in triplicate cultures. ChoB = housekeeping gene mitochondrial ribosomal protein S2; TLR = toll-like receptor.

Each figure legend includes the number of mice used in the experiments. All data were collected and analyzed by personnel blinded to the identity of the samples. Single data points are shown in Figs. 1-6 and Supplementary Fig. 1b,d,i with mean ± interquartile range (IQR). In Supplementary Fig. 1a,c,e-h data are shown as mean ± standard deviation (SD).

**Fig 7.**
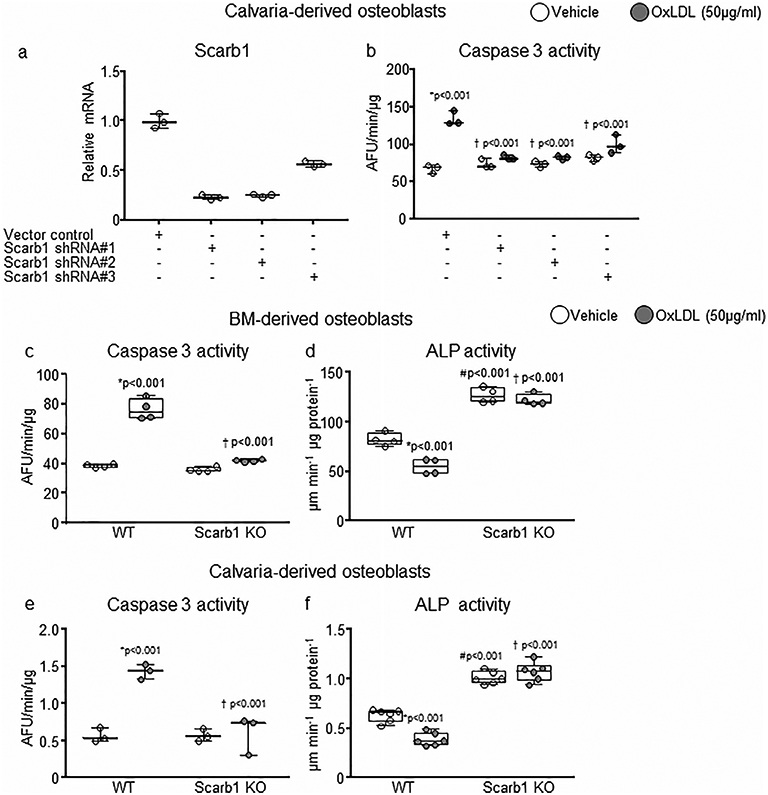
Scarb1 is required for the proapoptotic effects of OxPL on osteoblasts in culture. (*A*) Scarb1 expression was reduced with shRNA in calvaria-derived osteoblasts by 77% with shRNA#1, 75% with shRNA #2, and 43% with shRNa #3, respectively. Target gene expression was calculated as in *A*. (*B*) Caspase 3 activity was measured 6 hours after addition of vehicle or OxLDL (50 μg/mL) in the same cells as in *B*. Data analyzed by ANOVA, the *p* values shown are adjusted using the Holm-Sidak comparison procedure. *p < .001 versus respective vehicle; †*p* < .001 versus vector control with OxLDL. (*C-F*) Caspase 3 activity and alkaline phosphatase activity were measured, 6 hours or 2 days after addition of vehicle or OxLDL (50 μg/mL), respectively, in bone marrow-derived and calvaria-derived osteoblasts obtained from Scarb1 knockout mice. Bone marrow cells were harvested from 4-month-old to 5-month-old mice (*n* = 3/group) and calvaria from new born mice (WT *n* = 5, KO *n* = 2). Data analyzed by ANOVA, the *p* values shown are adjusted using the Holm-Sidak comparison procedure. *Versus respective vehicle; #*p* < .001 versus WT with vehicle; †*p* WT with OxLDL. Data are shown as box-plots with individual values and median and interquartile range. All measures were performed in triplicate cultures. AFU = arbitrary fluorescence units; ALP = alkaline phosphatase; KO = knockout.

Statistical analyses for the data shown in Figs. 1-6, Supplementary Fig. 1b,d, and Supplementary Fig. 3 were performed using GraphPad Prism (versions 7.0.4 and 8.0.1; GraphPad Software, Inc., La Jolla, CA, USA). Statistical analysis for the data shown in Fig. 7 was performed using SigmaPlot (versions 12.5; Systat Software, Inc., San Jose, CA, USA). Group mean values were compared by Student’s two-tailed *t* test, by ANOVA or ANOVA repeated measures as appropriate. When ANOVA indicated a significant effect, pairwise multiple comparisons were performed and the *p* values adjusted using the Tukey’s pairwise comparison procedure, the Holm-Sidak method, or the Bonferroni method as appropriate. Statistical analysis for the data shown in Supplementary Fig. 1a,c,e-h was performed using R (version 3.5; R Foundation for Statistical Computing, Vienna, Austria; https://www.r-project.org/). All of the calculated variables and statistical analyses for Figs. 1-4 and Supplementary Fig. 1b,d are presented in tabular form in the Supporting Information. The data on the supplementary tables are shown as mean ± SE as calculated in GraphPad Prism, which gives only SE.

For the in vivo studies, the sample size was adequate to detect a difference of 1.2 SD at a power of 0.8, and *p* < .05.^(17)^ For in vitro experiments, the number of replicates was sufficient to provide confidence in the measurements.

### Study approval

All animal procedures were approved by the Institutional Animal Care and Use Committees of the University of Arkansas for Medical Sciences.

## Results

### E06-scFv transgenic homozygous female and male mice are slightly smaller but appear equally healthy

WT, hemizygous, and homozygous mice were housed in the same vivarium and fed the same chow diet. All mice were healthy and did not display any obvious phenotypic or breeding differences. All mice gained weight throughout the 6-month observational period (Supplementary Fig. 1a,c,e-h). At 2 months of age, female mice displayed no difference in weight between the three genotypes (*p* > .5). However, at 6 months, hemizygous and homozygous females weighed 3.6% less (*p* = .09) and 7.1% less (*p* = .007), respectively, than female WT littermates. There was no difference in weight between hemizygous and homozygous females. These results indicate that, in females, the homozygous group had a slightly lower rate of weight gain compared to WT and hemizygous mice (details provided in the figure legend). In males, at 2 months of age, the homozygous mice weighed less than WT and hemizygous mice (7.7% less [*p* = .02] and 5.3% less [*p* = .08], respectively). However, at 6 months of age, there was no difference in weight in the males between the three genotypes, except for a trend for the homozygous mice to weigh less than WT (6.6%; *p* = .08). There was no difference in the percentage of fat mass and lean mass between WT, hemozygous, and homozygous females (Supplementary Fig. 1e,f) or males (Supplementary Fig. 1g,h) at any time point. In addition, there was no difference in the weight of vital organs such as liver, heart, spleen, and brain between WT and homozygous females at 7 months of age (Supplementary Fig. 1i). The weight of both kidneys was slightly increased in the homozygous mice.

At 6 months of age, femoral length was not different between the groups, except for a small decrease of 1.9% (*p* = .03) observed in female homozygous mice compared to WT (Supplementary Fig. 1, Supplementary Tables 3 and 4).

### E06-scFv increases trabecular bone in both females and male mice

We previously observed that the E06-scFV increases trabecular bone mass in homozygous males fed a normal diet.^(5)^ To determine if this bone anabolic effect was present also in females, and whether it was dependent on E06-scFv transgene dose, we compared 6-month-old homozygous, hemizygous, and WT littermates from both sexes. E06-scFv female mice, at 6 months of age, exhibited an increase in trabecular bone mass in the vertebra, femur, and tibia compared to WT mice (Fig. 1A-E). This effect was more pronounced in the homozygous than the hemizygous mice. Specifically, in the vertebra, the hemizygous and homozygous transgenic females showed a mean 16.7% (*p* < .0001) and 28.4% (*p* < .0001) increase in bone volume/total volume (BV/TV), respectively, compared to WT mice. In the femur, the mean increase in BV/TV for the hemizygous and homozygous transgenic females was 70.2% (*p* = .002) and 262.5% (*p* < .0001), respectively, compared to WT mice. In the tibia, the mean increase in BV/TV was 52.9% (*p* = .1070) and 218.9% (*p* < .001) in hemizygous and homozygous E06-scFv transgenic mice, compared to WT, respectively (Fig. 1, Supplementary Table 1).

The increase in BV/TV at all sites was associated with increased trabecular number and consequently reduced trabecular separation. Trabecular thickness was increased in the femur and tibia in the homozygous mice, but it was unchanged in the spine in both hemizygous and homozygous mice. Similar to previous observations in males,^(5)^ examination of the femur in the homozygous E06-scFv females revealed that the trabecular bone extended into the diaphyseal region, which is well beyond the metaphyseal location seen in the WT (Fig. 1C).

In the vertebra of the male mice, both homozygous and hemizygous mice had increased trabecular bone (9.2% [*p* = .0471] and 10.0% [*p* = .072] versus WT, respectively) (Fig. 2A). Homozygous, but not hemizygous, E06-scFv transgenic mice also exhibited an increase in trabecular bone at the femur and tibia (mean increase of 42.3% [*p* = .0130] and 64.9% [*p* < .0001] versus WT, respectively). These increments were smaller than the ones observed in females (Fig. 2A-C, Supplementary Table 2).

### E06-scFv increases cortical thickness in both female and male mice

Targeting OxPL increased cortical thickness in the femoral and tibial diaphysis in female homozygous mice, as compared to the WT littermates (Fig. 3A,B). The increase in cortical thickness was 8.2% (*p* < .0001) in the femur and 5.3% (*p* = .0377) in the tibia (Supplementary Table 3). This increase was associated, in both femur and tibia, with a decrease in medullary area (24% in the femur [*p* < .0001] and 12% in the tibia [*p* < .0001] in homozygous mice compared to WT), indicating an increase in endosteal bone apposition (Fig. 3A,B, Supplementary Table 3). In females, total area was also decreased in both homozygous and hemizygous mice in the femur and the tibia, indicating reduced periosteal apposition (Fig. 3A,B, Supplementary Table 3).

In males, there was an increase in cortical thickness at the diaphysis in both femur and tibia in homozygous mice of 7.6% (*p* = .0049) and 12.0% (*p* = .0019), respectively, as compared to the WT littermates (Fig. 3C,D, Supplementary Table 4). As in females, the increase in cortical thickness at both sites was due to a decrease in medullary area (14% in the femur [*p* = .0006] and 24% in the tibia [*p* < .0001] in homozygous mice compared to WT) (Fig. 3C,D, Supplementary Table 4). In males, however, total area was not changed in the three groups (Fig. 3C,D, Supplementary Table 4).

### E06-scFv increases bone mass by increasing bone formation in trabecular and cortical bone

Histomorphometric analysis of the trabecular bone of the vertebrae in E06-scFv homozygous female mice showed a 58% increase in osteoblast number (*p* = .0003), and a 36% decrease in osteoclast numbers (*p* = .000001) (Fig. 4A, Supplementary Table 5). Mineralizing surface per bone surface increased by 59% (*p* = .0007) and bone formation rate by 98% (*p* = .0005) in homozygous female mice compared to WT (Fig. 4A). Similarly, in the trabecular bone of the femur there was a 60% increase in mineralizing surface per bone surface (*p* = .015) and a 110% increase in bone formation rate (*p* = .0011) (Fig. 4B, Supplementary Table 5). At this site, however, we did not detect a change in osteoblast or osteoclast number. Representative histomorphometric images of the trabecular bone of the femur are shown in Supplementary Fig. 2. In the endocortical surface of the femur, there was a 46% increase in osteoblast number (*p* = 0.035) and a 56% increase in bone formation rate (*p* = .014) (Fig. 4C, Supplementary Table 5). Osteoclast number was unchanged. There was no evidence of calcified cartilage in either WT or transgenic mice. Collectively, these data indicate that osteoblasts are likely a major cell target of the anabolic effect of E06-scFv in both the trabecular and the cortical bone compartments.

### E06-scFv transgenic mice have circulating antibody levels sufficient to prevent the pro-apoptotic effect of OxLDL on osteoblastic cells in vitro and in vivo

The E06-scFv transgene is under the control of the ApoE promoter, which is primarily expressed in hepatocytes and macrophages.^(3)^ We quantified the relative serum levels of the E06-scFv by ELISA in the various cohorts. In both male and female mice, there was a significant gene-dose effect with higher E06-scFv levels in homozygous compared to hemizygous mice (Fig. 5A).

We then compared the ability of serum from WT, homozygous, and hemizygous mice to block the adverse effects of OxPL in cell culture, using OxLDL as a well-documented biological source of OxPL. As shown in Fig. 5B, serum from E06-scFv homozygous female mice protected cultured osteoblastic cells from OxLDL-induced apoptosis at each dilution examined, whereas serum from WT mice did not. The serum from hemizygous mice had an intermediate effect. Moreover, measurement of osteoblast apoptosis in vivo, quantified by TUNEL staining in vertebral bone, was decreased in homozygous E06-scFv transgenic mice, confirming that decreased apoptosis is a mechanism by which neutralizing OxPL enhances bone anabolism in vivo (Fig. 5C).

### Scarb1 is required for the pro-apoptotic effects of OxPL on osteoblasts in culture

In spite of the in vitro evidence for decreased osteoclastogenesis (Supplementary Fig. 3), our histomorphometric analysis strongly suggested that the anabolic effect of targeting OxPL was due largely to enhanced osteoblast number and function. We therefore further investigated in vitro whether the adverse effects of OxPL on bone mass might be due to direct actions on osteoblasts.^(15,18-23)^ OxPL are specifically recognized by the scavenger receptors CD36 and Scarb1, and by TLR2, TLR4, and TLR6.^(2)^ Moreover, OxPL promote apoptosis in multiple cell types. In particular, OxPL-induced apoptosis of mouse peritoneal macrophages is mediated by a CD36-TLR2–dependent mechanism.^(24)^ Gene expression analysis by qPCR showed that Scarb1 was prominently expressed in osteoblast-like and osteocyte-like cell lines, as well as in calvaria-derived osteoblasts. In contrast, CD36 expression was very low or undetectable and TLRs were expressed at a much lower level (Fig. 6A,B). Compared to Scarb1, TLR2 and TLR4 expression was 66% to 94% lower and TLR6 expression was 88% to 99% lower in the cell types examined. In addition, Scarb1 expression was decreased in osteoblasts of homozygous E06-scFv transgenic mice (Fig. 6C).

It has been previously reported that Scarb1 knockout mice have increased bone mass, consistent with the notion that this SR might mediate pro-apoptotic effects of OxPL on osteoblasts.^(7,25)^ OxPL are a prominent component of OxLDL and are known to mediate uptake and adverse effects of OxLDL in macrophages in vitro and in vivo.^(1,4)^ We therefore examined whether Scarb1 could be involved in the adverse effect of OxLDL on osteoblasts. Silencing Scarb1 with shRNA protected the calvaria-derived osteoblasts from OxLDL-induced apoptosis (Fig. 7A,B). Moreover, both bone marrow–derived and calvaria-derived osteoblasts from Scarb1 knockout mice were protected from the pro-apoptotic (Fig. 7C,E) and anti-differentiating effects of OxLDL (Fig. 7D,F). These results strongly suggest that SR Scarb1 is primarily responsible for mediating the deleterious effect of OxPL/OxLDL on osteoblastic cells in vitro.

## Discussion

We previously demonstrated that neutralizing OxPL by expressing the E06-scFv antibody in high cholesterol and western-diet-fed *Ldlr^−/−^* mice prevented the adverse effects of hyperlipidemia on bone mass.^(5)^ This suggested that OxPL restrain bone formation in the hyperlipidemic setting, in which lipid peroxidation is greatly exaggerated and OxPL are abundantly produced in tissues. In the present study, we show that expression of the E06-scFv antibody in C57BL/6J mice fed a regular chow diet increased both trabecular and cortical bone in 6-month-old male and female mice. Notably, the increase in bone mass was more pronounced in homozygous mice, indicating a dose-dependent effect of the transgene in vivo. The increase in trabecular bone mass was seen in the spine, distal femur, and proximal tibia of male and female mice. Similarly, the increase in cortical bone was present in both the femur and tibia of both sexes. These results suggest that, under physiologic conditions, OxPL restrain bone formation, at least up to early adulthood and perhaps through all stages of life.

The source of OxPL during bone growth and development is unknown. Cells undergoing apoptosis are enriched in OxPL^(26)^ and so are the variety of apoptotic bodies, extracellular vesicles and microparticles released from cells that are oxidatively stressed and/or undergoing apoptosis.^(2,27)^ Conceivably, the rapid growth and the accompanying high cell turnover that occurs during bone development are responsible for the increased generation and release of OxPL.^(28,29)^ However, it remains possible that OxPL are produced in sufficiently high enough levels in the bone microenvironment throughout life and therefore chronically restrain bone formation. This may be a factor for the development of osteoporosis in old age, when oxidative stress is a culprit.^(30,31)^

The increased bone mass in the E06-scFv transgenic mice was associated with an increase in osteoblast number and bone formation, in both the trabecular and the cortical bone compartments. Additionally, we found a decrease in osteoblast apoptosis in cancellous bone in transgenic homozygous mice in vivo. These results support the possibility that a major or even predominant mechanism of the adverse effect of OxPL is promotion of osteoblast apoptosis. Hence, the anabolic effect of E06-scFv appears to relate to the ability to block the adverse effects of OxPL on osteoblasts and to facilitate increased osteoblast number. This interpretation is in agreement with our previous studies showing that attenuation of apoptosis in cells of the osteoblast lineage increases cancellous bone mass.^(32)^ However, OxPL have diverse effects on different cell types and act via a variety of receptor-mediated, as well as non-receptor-mediated mechanisms.^(2-4)^ Thus, their impact on bone metabolism is likely to be complex and an effect on osteoblast differentiation cannot be excluded. Whatever the precise mechanisms, our data clearly demonstrate an unexpected negative effect of OxPL during normal bone growth in both male and female mice. Our observations, therefore, suggest that targeting OxPL maybe a novel therapeutic target to effect enhanced bone anabolism under physiological conditions.

We have previously shown that both CD36 and Scarb1 specifically recognize OxPL.^(2)^ Additionally, we and others have demonstrated that OxPL decrease the proliferation, differentiation, and survival of osteoblastic cells.^(18-23)^ Moreover, many adverse effects of OxPL are prevented by the E06 IgM and the E06-scFv transgene.^(5)^ Scarb1 and CD36, the two major scavenger receptors for OxPL, are expressed in osteoblasts and have been implicated in the uptake of OxLDL, cholesteryl ester, and estradiol by this cell type.^(25)^ Further, global deletion of Scarb1 results in enhanced bone formation.^(7)^ Therefore, CD36 and Scarb1 were a priori likely candidates as mediators of the negative effects OxPL on osteoblasts. In support of this scenario, we show in the present work that Scarb1 is expressed by osteoblastic cells, and that silencing or absence of Scarb1 blocks the OxLDL-induced increase of osteoblast apoptosis and decrease differentiation of osteogenic precursors. These results strongly suggest that, at least in vitro, other OSE receptors such as TLRs cannot compensate for the lack of Scarb1 in osteoblastic cells. Therefore, Scarb1 must be an essential mediator of the direct effects of OxPL on osteoblasts.

Consistent with our findings, mice with germline deletion of Scarb1 have increased osteoblast number, bone formation rate, and high bone mass.^(7,33,34)^ This phenotype, however, was attributed to the decreased uptake of high-density lipoprotein (HDL)-bound cholesterol by the adrenal glands and thereby defective glucocorticoid synthesis and elevated adrenocorticotropic hormone (ACTH) in the knockout mice.^(7)^ HDL-dependent regulation of caveolin gene expression in osteoblasts has also been proposed as an alternative explanation of the bone phenotype.^(7)^ Martineau and colleagues,^(33)^ on the other hand, have proposed that Scarb1 is not required for the suppression of bone marrow stromal cell number by OxLDL. However, in that study the investigators used much higher doses of a different OxLDL preparation than the one we used, and examined only cell number—not apoptosis or proliferation. In contrast, others have recently shown that Scarb1 knockout mice have lower bone mass and lower bone formation rate at 16 weeks, suggesting that Scarb1 is required for osteoblast differentiation and bone acquisition.^(35)^ Be that as it may, the global nature of the Scarb1 deletion in these later mouse models cannot exclude the possibility that other cell types contribute to the bone phenotype. Last, based on in vitro observations, recent studies suggested that OxLDL and synthetic OxPL may promote endocytosis of LRP6—a Wnt receptor that plays a prominent role in osteoblastogenesis.^(36)^ The relevance of these in vitro observations to the in vivo phenotype we report herein is, however, questionable because low-density lipoprotein receptor-related protein 6 (LRP6) deletion causes a decrease, not an increase in bone mass.^(37)^

In addition to the histologic demonstration of increased osteoblast numbers and bone formation rate in our E06-scFv transgenic mice, we found a decrease in osteoclast number in trabecular bone of the vertebra and decreased osteoclastogenesis in vitro. This finding raises the possibility that OxPL may promote osteoclast activity during physiologic growth. Albeit, osteoclast number was unchanged in the cortical and trabecular bone of the femur. These results are also in agreement with our previous work showing a decrease in osteoclast numbers in vertebral bone of 11-month-old mice fed a normal diet, but no changes in osteoclast number in the trabecular or cortical bone of the femur of E06-scFv transgenic mice fed a high-fat diet.^(5)^ The role of OxPL on osteoclastogenesis is unclear and previous studies have yielded conflicting results. Indeed, some reports suggested that high-fat diet and OxLDL inhibit osteoclastogenesis,^(17,38)^ whereas others found that OxLDL promotes osteoclastogenesis.^(39-41)^

The reason why osteoclasts decreased only in the trabecular bone of the vertebra, but not the trabecular or cortical bone of the femur is not known. Nevertheless, in several other settings osteoclasts behave differently in separate bone compartments. For example, glucocorticoid excess does not change the number of osteoclasts in trabecular bone,^(42)^ but increases osteoclasts in cortical bone.^(43)^ Similarly, in aged mice, osteoclasts are decreased in trabecular bone,^(44)^ but increased on the endosteal surface of the femur.^(45,46)^ Furthermore, deletion of RANKL in osteoblasts and osteocytes increases bone mass in trabecular, but not cortical bone^(47)^; and Wnt5a increases bone resorption and decreases trabecular, but not cortical bone.^(48)^

In homozygous E06-scFv transgenic female, but not male, mice there was a small decrease in femoral length and total bone area compared to WT littermates; albeit, these mice had increased bone formation at the endosteal surface. We have no explanation for this seemingly discrepancy, but we think it is unlikely that OxPL have an inhibitory effect on the endosteal surface, but a stimulatory effect on the periosteal surface. The reduced bone size can only be explained by reduced periosteal apposition. A plausible explanation is that decreased mechanical loading, resulting from lower body weight has influenced periosteal apposition; but such relationships have yet to be established and the cause of the slightly lower body weight in E06-scFv females remains to be determined. Future work, perhaps in growing mice when growth is easily measured or in mice subjected to mechanical loading, will be necessary to understand this unexpected effect of the E06-scFV transgene.

In conclusion, the evidence described herein strongly suggests that OxPL restrain normal bone anabolism in both cortical and trabecular bone, at least during the first 6 months of life in male and female mice. The adverse effects of OxPL in bone homeostasis likely result from their ability to decrease osteoblast number and function, and perhaps increase osteoclast function at some sites. In addition, our findings indicate that OxPL promote osteoblast apoptosis and reduce osteoblast differentiation from precursors via Scarb1. E06, therefore, stimulates bone anabolism in healthy young mice, at least in part, by neutralizing OxPL and blocking their pro-apoptotic effects on mature osteoblasts and/or their precursors. For this reason, targeting OxPL by EO6 provides proof of principle for a novel therapeutic approach to bone anabolism.

## Supplementary Material

SUPPLEMENTARY TABLES

SUPPLEMENTARY FIGURES
