# Supplementary material for: A Neutralizing Antibody Targeting Oxidized Phospholipids Promotes Bone Anabolism in Chow-Fed Young Adult Mice
Source: J Bone Miner Res. Author manuscript; Available in PMC 2021 Feb 3. (PMC7855899; doi:10.1002/jbmr.4173)
Supplement: SUPPLEMENTARY TABLES [file NIHMS1647728-supplement-SUPPLEMENTARY_TABLES.docx]

**Supplementary Table 1: MicroCT parameters of trabecular bone in female mice**

| **Parameters**  **Females** | **WT**  **mean** | **Hemizygous**  **mean** | **Homozygous**  **mean** | **% change WT vs Hemi** | **SE**  **WT vs Hemi** | **p**  **WT vs Hemi** | **% change WT vs Homo** | **SE**  **WT vs Homo** | **p**  **WT vs Homo** | **% change Hemi vs Homo** | **SE**  **Hemi Vs Homo** | **p**  **Hemi vs Homo** |
| --- | --- | --- | --- | --- | --- | --- | --- | --- | --- | --- | --- | --- |
| **Vertebra** | | | | | | | | | | | | |
| **BV/TV** | 29.82% | 34.81% | 38.30% | **16.73** | 2.85 | <0.0001 | **28.44** | 3.79 | <0.0001 | **10.03** | 2.96 | 0.0036 |
| **Tb number** | 3.67/mm | 4.21/mm | 4.45/mm | **14.51** | 2.26 | <0.0001 | **21.15** | 3.00 | <0.0001 | **5.80** | 2.39 | 0.0477 |
| **Tb Thickness** | 0.071 mm | 0.069 mm | 0.070 mm | **-2.80** | 1.58 | 0.1869 | **-1.66** | 2.09 | 0.7086 | **1.18** | 1.97 | 0.8214 |
| **Tb separation** | 0.259 mm | 0.228 mm | 0.207 mm | **-13.9** | 2.38 | <0.0001 | **-19.73** | 3.16 | <0.0001 | **-6.69** | 3.34 | 0.1212 |
| **Femur (trabecular bone)** | | | | | | | | | | | | |
| **BV/TV** | 4.75% | 8.08% | 17.20% | **70.22** | 19.43 | 0.002 | **262.49** | 24.73 | <0.0001 | **112.95** | 12.56 | <0.0001 |
| **Tb number** | 2.91/mm | 3.24/mm | 3.77/mm | **11.05** | 2.45 | 0.0001 | **29.38** | 3.24 | <0.0001 | **16.50** | 2.66 | <0.0001 |
| **Tb Thickness** | 0.044 mm | 0.048 mm | 0.056 mm | **6.73** | 3.21 | 0.0992 | **28.53** | 4.25 | <0.0001 | **20.42** | 3.63 | <0.0001 |
| **Tb separation** | 0.344 mm | 0.305mm | 0.251 mm | **-11.28** | 2.12 | <0.0001 | **-26.87** | 2.80 | <0.0001 | **-17.57** | 2.88 | <0.0001 |
| **Tibia (trabecular bone)** | | | | | | | | | | | | |
| **BV/TV** | 6.96% | 10.65% | 22.21% | **52.93** | 25.11 | 0.107 | **218.93** | 25.11 | <0.0001 | **108.54** | 16.42 | <0.0001 |
| **Tb number** | 3.04/mm | 3.50/mm | 4.33/mm | **15.28** | 5.92 | 0.04 | **45.52** | 5.92 | <0.0001 | **23.62** | 5.13 | 0.0003 |
| **Tb Thickness** | 0.055 mm | 0.058 mm | 0.069 mm | **5.83** | 4.38 | 0.39 | **25.98** | 4.38 | <0.0001 | **19.03** | 4.14 | 0.0003 |
| **Tb separation** | 0.332 mm | 0.282 mm | 0.225 mm | **-15.01** | 4.17 | 0.0035 | **-32.44** | 4.17 | <0.0001 | **-20.50** | 4.90 | 0.0008 |

Mean, % change, standard error of the change and significance between groups of the indicated micro-CT parameters in WT, hemizygous and homozygous female mice. Data relative to Figure 1. BV/TV: bone volume/total volume; Tb: trabecular.

**Supplementary Table 2: MicroCT parameters of trabecular bone in male mice**

| **Parameters**  **Males** | **WT**  **mean** | **Hemizygous**  **mean** | **Homozygous**  **mean** | **% change WT vs Hemi** | **SE**  **WT vs Hemi** | **p**  **WT vs Hemi** | **% change WT vs Homo** | **SE**  **WT vs Homo** | **p**  **WT vs Homo** | **% change Hemi vs Homo** | **SE**  **Hemi Vs Homo** | **p**  **Hemi vs Homo** |
| --- | --- | --- | --- | --- | --- | --- | --- | --- | --- | --- | --- | --- |
| **Vertebra** | | | | | | | | | | | | |
| **BV/TV** | 32.97% | 36.00 % | 36.28% | **9.19** | 3.78 | 0.0471 | **10.04** | 4.54 | 0.0782 | **0.77** | 3.45 | 0.9724 |
| **Tb number** | 4.77//mm | 4.99/mm | 4.99/mm | **4.59** | 2.13 | 0.0896 | **4.67** | 2.56 | 0.1411 | **0.08** | 1.70 | 0.9988 |
| **Tb Thickness** | 0.065 mm | 0.066 mm | 0.066 mm | **0.78** | 3.16 | 0.9657 | **0.61** | 3.77 | 0.9855 | **-0.18** | 3.24 | 0.9983 |
| **Tb separation** | 0.186 mm | 0.177 mm | 0.179 mm | **-4.68** | 2.47 | 0.1509 | **-3.93** | -3.00 | 0.3963 | **0.79** | 2.50 | 0.9466 |
| **Femur (trabecular bone)** | | | | | | | | | | | | |
| **BV/TV** | 12.05% | 13.77% | 17.15% | **14.27** | 11.99 | 0.4639 | **42.32** | 14.39 | 0.013 | **24.54** | 10.52 | 0.0593 |
| **Tb number** | 4.14/mm | 4.31/mm | 4.41/mm | **4.20** | 2.69 | 0.2700 | **6.63** | 3.23 | 0.1091 | **2.32** | 2.59 | 0.6447 |
| **Tb Thickness** | 0.051 mm | 0.049 mm | 0.052 mm | **-2.18** | 4.27 | 0.866 | **3.01** | 5.13 | 0.8281 | **5.31** | 4.37 | 0.4501 |
| **Tb separation** | 0.232 mm | 0.222 mm | 0.219 mm | **-4.44** | 3.16 | 0.3465 | **-5.69** | 3.76 | -.2931 | **-1.30** | 3.20 | 0.9122 |
| **Tibia (trabecular bone)** | | | | | | | | | | | | |
| **BV/TV** | 15.15% | 18.05% | 24.98% | **19.14** | 11.76 | 0.2476 | **64.88** | 11.78 | <0.0001 | **38.39** | 9.70 | 0.0010 |
| **Tb number** | 4.52/mm | 4.61/mm | 4.88/mm | **1.97** | 3.48 | 0.8391 | **7.96** | 3.48 | 0.0707 | **5.87** | 3.34 | 0.1986 |
| **Tb** **Thickness** | 0.059 mm | 0.061 mm | 0.068 mm | **4.47** | 4.52 | 0.5886 | **16.15** | 4.52 | 0.0029 | **11.18** | 4.21 | 0.0323 |
| **Tb separation** | 0.207 mm | 0.202 mm | 0.189 mm | **-2.65** | 4.41 | 0.8204 | **-8.97** | 4.41 | 0.1192 | **-6.49** | 4.44 | 0.3213 |

Mean, % change, standard error of the change and significance between groups of the indicated micro-CT parameters in WT, hemizygous and homozygous male mice. Data relative to Figure 2. BV/TV, bone volume/total volume. Tb, trabecular.

**Supplementary Table 3: MicroCT parameters of cortical bone in female mice**

| **Parameters**  **Females** | **WT**  **Mean** | **Hemizygous**  **Mean** | **Homozygous**  **Mean** | **% change WT vs Hemi** | **SE**  **WT vs Hemi** | **p**  **WT vs Hemi** | **% change WT vs Homo** | **SE**  **WT vs Homo** | **p**  **WT vs Homo** | **% change Hemi vs Homo** | **SE**  **Hemi Vs Homo** | **p**  **Hemi vs Homo** |
| --- | --- | --- | --- | --- | --- | --- | --- | --- | --- | --- | --- | --- |
| **Femur (cortical bone)** | | | | | | | | | | | | |
| **Cortical thickness** | 0.220 mm | 0.226 mm | 0.238 mm | **2.64** | 1.21 | 0.0843 | **8.18** | 1.61 | <0.0001 | **5.401** | 1.43 | 0.0012 |
| **Total area** | 1.766 mm^2^ | 1.646 mm^2^ | 1.535 mm^2^ | **-6.79** | 1.41 | <0.0001 | **-13.08** | 1.85 | <0.0001 | **-6.74** | 1.82 | 0.0014 |
| **Medullary area** | 0.890 mm^2^ | 0.791 mm^2^ | 0.677 mm^2^ | **-11.12** | -1.81 | <0.0001 | **-23.98** | 2.39 | <0.0001 | **-14.47** | 2.45 | <0.0001 |
| **Femoral length** | 16.18 mm | 15.99 mm | 15.87 mm | **-1.17** | 0.56 | 0.1014 | **-1.91** | 0.75 | 0.0346 | **-0.75** | 0.70 | 0.5336 |
| **Tibia (cortical bone)** | | | | | | | | | | | | |
| **Cortical thickness** | 0.242 mm | 0.244 mm | 0.254 mm | **1.03** | 2.03 | 0.8674 | **5.30** | 2.03 | 0.0377 | **4.22** | 2.01 | 0.1089 |
| **Total area** | 0.938 mm^2^ | 0.846 mm^2^ | 0.828 mm^2^ | **-9.78** | 2.10 | 0.0002 | **-11.77** | 2.10 | <0.0001 | **2.20** | 2.32 | 0.6173 |
| **Medullary area** | 0.280 mm^2^ | 0.230 mm^2^ | 0.207 mm^2^ | **-17.71** | 4.52 | 0.0015 | **-25.90** | 4.52 | <0.0001 | **-9.96** | 5.50 | 0.1854 |

Mean, % change, standard error of the change and significance between groups of the indicated micro-CT parameters in WT, hemizygous and homozygous female mice. Data relative to Figure 3 a-b.

**Supplementary Table 4: MicroCT parameters of cortical bone in male mice**

| **Parameters**  **Males** | **WT**  **Mean** | **Hemizygous**  **Mean** | **Homozygous**  **Mean** | **% change WT vs Hemi** | **SE**  **WT vs Hemi** | **p**  **WT vs Hemi** | **% change WT vs Homo** | **SE**  **WT vs Homo** | **p**  **WT vs Homo** | **% change Hemi vs Homo** | **SE**  **Hemi Vs Homo** | **p**  **Hemi vs Homo** |
| --- | --- | --- | --- | --- | --- | --- | --- | --- | --- | --- | --- | --- |
| **Femur (cortical bone)** | | | | | | | | | | | | |
| **Cortical thickness** | 0.206 mm | 0.214 mm | 0.221 mm | **4.13** | 1.93 | 0.0901 | **7.58** | 2.31 | 0.0049 | **3.31** | 1.85 | 0.1811 |
| **Total area** | 1.900 mm^2^ | 1.849 mm^2^ | 1.783 mm^2^ | **-2.68** | 2.58 | 0.5553 | **-6.16** | 3.08 | 0.1228 | **-3.57** | 2.64 | 0.3737 |
| **Medullary area** | 1.027 mm^2^ | 0.954 mm^2^ | 0.881mm^2^ | **-7.06** | 2.99 | 0.0558 | **-14.16** | 3.58 | 0.0006 | **-7.64** | 3.21 | 0.0534 |
| **Femoral length** | 15.82 mm | 15.87 mm | 15.85 mm | **0.32** | 0.63 | 0.8726 | **0.19** | 0.89 | 0.9753 | **-0.13** | 0.52 | 0.9684 |
| **Tibia (cortical bone)** | | | | | | | | | | | | |
| **Cortical thickness** | 0.228 mm | 0.240 mm | 0.255 mm | **5.80** | 3.16 | 0.173 | **12.08** | 3.23 | 0.0019 | **5.94** | 3.01 | 0.1342 |
| **Total area** | 1.052 mm^2^ | 1.034 mm^2^ | 0.989 mm^2^ | **-1.71** | 3.92 | 0.9007 | **-6.00** | 3.94 | 0.2936 | **-4.36** | 3.91 | 0.5113 |
| **Medullary area** | 0.375 mm^2^ | 0.332 mm^2^ | 0.285 mm^2^ | **-11.46** | 4.03 | 0.0200 | **-23.99** | 4.12 | <0.0001 | **-14.15** | 4.57 | 0.0106 |

Mean, % change, standard error of the change and significance between groups of the indicated micro-CT parameters in WT, hemizygous and homozygous male mice. Data relative to Figure 3 c-d.

**Supplementary Table 5: Histomorphometric parameters of cancellous and cortical bone in female mice**

| **Parameters**  **Females** | **WT**  **Mean ± SEM** | **Homozygous**  **Mean ± SEM** | **% change ± SEM** | **p** |
| --- | --- | --- | --- | --- |
| **Vertebra** | | | | |
| **Ob.N/B.Pm** | 5.914 ± 0.307 /mm | 9.328 ± 0.5411 /mm | **57.73 ± 10.80** | <0.0001 |
| **Oc.N/B.Pm** | 0.784 ± 0.027 /mm | 0.506± 0.032 /mm | **-34.62 ± 5.14** | <0.0001 |
| **MS/BS** | 18.580 ± 0.942 % | 29.500 ± 2.443 % | **58.77 ± 13.39** | 0.0007 |
| **MAR** | 1.152 ± 0.035 µm/day | 1.423 ± 0.047 µm/day | **23.52 ± 5.01** | 0.0004 |
| **BFR/BS** | 0.2152 ± 0.015 µm^2^ µm^-1^ day^-1^ | 0.4254 ± 0.046 µm^2^ µm^-1^ day^-1^ | **97.67 ± 21.08** | 0.0005 |
| **Femur (trabecular bone)** | | | | |
| **Ob.N/B.Pm** | 14.560 ± 1.616 /mm | 15.87 ± 1.651 /mm | **8.99 ± 18.47** | 0.5784 |
| **Oc.N/B.Pm** | 2.109 ± 0.225 /mm | 1.928 ± 0.273 /mm | **-8.58 ± 16.58** | 0.6103 |
| **MS/BS** | 21.180 ± 3.001 % | 33.210 ± 3.102 % | **59.80 ± 20.41** | 0.0156 |
| **MAR** | 1.098 ± 0.066 µm/day | 1.555 ± 0.098 µm/day | **41.62 ± 0.12** | 0.0016 |
| **BFR/BS** | 0.243 ± 0.045 µm^2^ µm^-1^ day^-1^ | 0.510 ± 0.046 µm^2^ µm^-1^ day^-1^ | **110.10 ± 26.39** | 0.0011 |
| **Femur (endosteal surface)** | | | | |
| **Ob.N/B.Pm** | 5.221 ± 0.649 /mm | 7.601 ± 0.854 /mm | **45.5 ± 20.18** | 0.0354 |
| **Oc.N/B.Pm** | 0.646 ± 0.044 /mm | 0.696 ± 0.062 /mm | **7.74 ± 11.70** | 0.5160 |
| **MS/BS** | 28.350 ± 3.784 % | 34.220 ± 5.566 % | **20.71 ± 16.64** | 0.2354 |
| **MAR** | 0.845 ± 0.032 | 1.103 ± 0.034 | **30.53 ± 5.60** | 0.0001 |
| **BFR/BS** | 0.241 ± 0.036 µm^2^ µm^-1^ day^-1^ | 0.378 ± 0.032 µm^2^ µm^-1^ day^-1^ | **56.35 ± 20.06** | 0.0148 |

Mean, standard error, % change and significance of the indicated histomorphometric parameters between WT and homozygous female mice. Data relative to Figure 4. Ob.N, osteoblast number. B.Pm, bone perimeter. Oc.N, osteoclast number. MS, mineralized surface. BS, bone surface. MAR, mineral apposition rate. BFR, bone formation rate.

**Supplementary Table 6. TaqMan assays used for quantification of mRNA by qPCR**

| **NCBI gene name** | **Gene name** | **TaqMan #** |
| --- | --- | --- |
| **Scarb1** | scavenger receptor class B, member 1 | Mm00450234_m1 |
| **CD36** | CD36 antigen | Mm00432403_m1 |
| **TLR2** | toll-like receptor 2 | Mm00442346_m1 |
| **TLR4** | toll-like receptor 4 | Mm00445273_m1 |
| **TLR6** | toll-like receptor 6 | Mm02529782_s1 |

List of the TaqMan primers used in qPCR assays
