# Supplementary material for: A Neutralizing Antibody Targeting Oxidized Phospholipids Promotes Bone Anabolism in Chow-Fed Young Adult Mice
Source: J Bone Miner Res. Author manuscript; Available in PMC 2021 Feb 3. (PMC7855899; doi:10.1002/jbmr.4173)
Supplement: SUPPLEMENTARY FIGURES [file NIHMS1647728-supplement-SUPPLEMENTARY_FIGURES.docx]

**
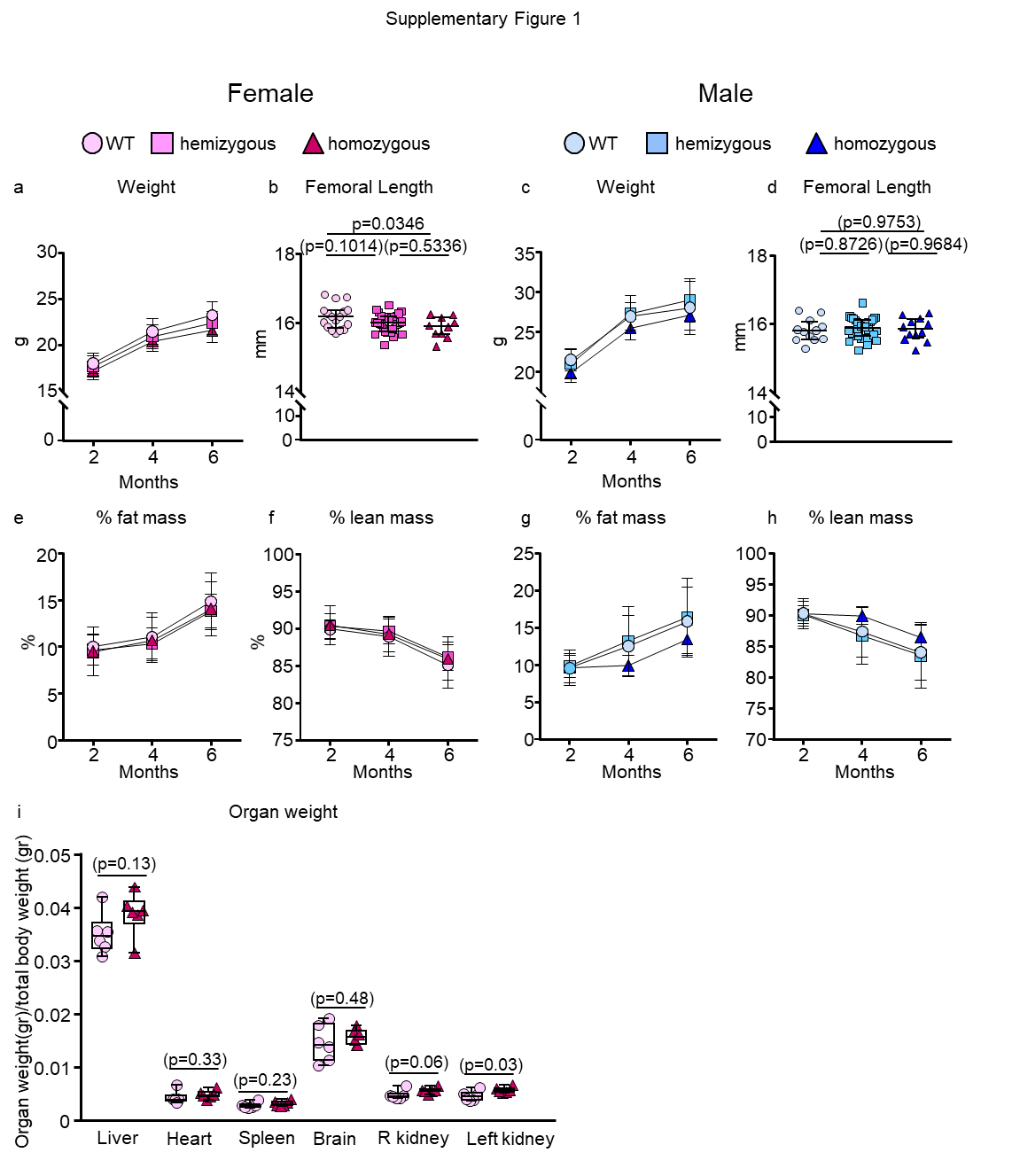
**

**Supplementary Figure 1. E06-scFv expression decreases the weight in homozygous and hemizygous females and in homozygous males.**

Weight measurements at 2, 4 and 6 months of age in **a)** female and in **c)** male mice. Female: WT n=17; hemizygous E06-scFv n=32; homozygous E06-scFv n=10. Male: WT n=13; hemizygous E06-scFv n=34; homozygous E06-scFv n=13. The measurements of total weight, by ANOVA repeated measures, indicated differences in rate of weight gain between the genotypes in females (p=0.007085) and males (p=0.01671). Further analysis, showed that the fastest rate of weight gain was in females in WT (1.72 and 0.87 g/month in the first 2 months and in the last two months respectively) whereas the homozygous had the slowest rate of weight gain (1.60 and 0.6 gr/month). The average gram difference considering every time point was 0.6 grams between WT and hemizygous mice (p=0.02), 1.2 gr between WT and homozygous mice (p<0.0001) and 0.59 gr between homozygous and hemizygous mice (p=0.06). In males, the WT weight gain rate was the slowest (2.75 and 0.53 gr/month) whereas the hemizygous rate was the highest (3.17 and 0.86 gr/month). The average gram difference considering every time point was 0.2 grams between WT and hemizygous mice (p=1), 1.3 gr between WT and homozygous mice (p=0.01) and 1.6 gr between homozygous and hemizygous mice (p=0.0001). Data are shown as mean and standard deviation. Femoral lenght for WT, hemizygous and homozygous female and male mice is shown in **b)** and **d)** respectively. Female: WT n=17; hemizygous E06-scFv n=31; homozygous E06-scFv n=10. Male: WT n=12; hemizygous E06-scFv n=32; homozygous E06-scFv n=12. Data are shown as individual values and median with interquartile range. Data analyzed by ANOVA, the shown P values are adjusted using the Tukey’s pairwise comparison procedure. **e,f)** Percentage of fat mass and lean mass evaluated by DXA BMD in female mice at 2,4,6 months of age. There was no change between phenotypes (p= 0.2767) by ANOVA repeated measures. Female: WT n=17; hemizygous E06-scFv n=32; homozygous E06-scFv n=10. Data are shown as mean and standard deviation **g,h)** Percentage of fat mass and lean mass in male mice at 2,4,6 months of age. There was no change between phenotypes (p=0.1614) by ANOVA repeated measures. Male: WT n=13; hemizygous E06-scFv n=34; homozygous E06-scFv n=13. Data are shown as mean and standard deviation. **i)** Organ weight normalized per body weight in WT and homozygous female mice at 7 months of age. WT n=7; homozygous E06-scFv n=7. Data are shown as box plots and individual values and median with interquartile range. Data analyzed by Student t-test.

**
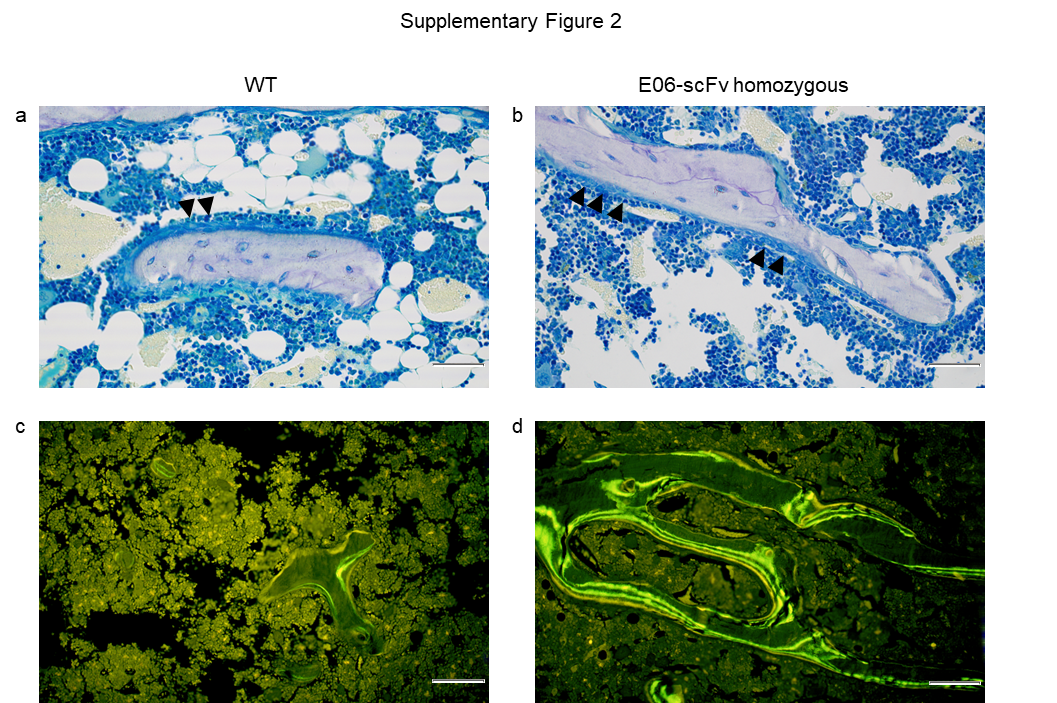
**

**Supplementary Figure 2.** E06-scFv affects bone mass by increasing osteoblasts in trabecular and cortical bone **a,b)** Longitudinal section of the femur stained for toluidin blue to visualize osteoblasts (black arrowheads). Magnification 40X, scale 50 µm. **c,d)** Unstained longitudinal sections at the distal methaphysis to visualize the calcein fluorochrome. Magnification 20X, scale 100 µm.


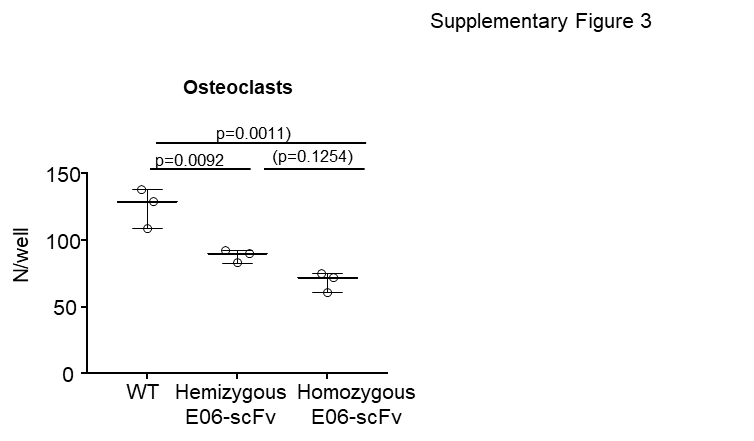


**Supplementary Figure 3.** Number of TRAP-positive multinucleated osteoclasts derived from bone macrophages of mice of the indicated genotypes cultured with M-CSF (30 ng/mL) and RANKL (30 ng/mL) for 5 days (n= 3 mice per group). Data are shown as box plots with individual values and median and interquartile range. Data analyzed by ANOVA, the shown p values are adjusted using the Tukey’s pairwise comparison procedure.
